# Interpleural location of chest drain on ultrasound excludes pneumothorax and associates with a low degree of chest drain foreshortening on the antero-posterior chest X-ray

**DOI:** 10.1186/s13089-022-00296-0

**Published:** 2022-11-17

**Authors:** Michal Maly, Masego Candy Mokotedi, Eva Svobodova, Marek Flaksa, Michal Otahal, Zdenek Stach, Jan Rulisek, Tomas Brozek, Michal Porizka, Martin Balik

**Affiliations:** 1 Department of Anesthesiology and Intensive Care, 1st Faculty of Medicine, Charles University and General University Hospital in Prague. U Nemocnice 2, Prague 2, 128 00 Prague, Czechia; 2 Department of Radiology, Institute of Clinical and Experimental Medicine, Videnska 1958/9, Prague 4, 14021 Prague, Czechia

**Keywords:** Pneumothorax, Chest ultrasound, Chest X-ray, Acute respiratory distress syndrome, Barotrauma, Covid-19

## Abstract

**Background:**

The role of chest drain (CD) location by bedside imaging methods in the diagnosis of pneumothorax has not been explored in a prospective study yet.

**Methods:**

Covid-19 ARDS patients with pneumothorax were prospectively monitored with chest ultrasound (CUS) and antero-posterior X-ray (CR) performed after drainage in the safe triangle. CD foreshortening was estimated as a decrease of chest drain index (CDI = length of CD in chest taken from CR/depth of insertion on CD scale + 5 cm). The angle of inclination of the CD was measured between the horizontal line and the CD at the point where it enters pleural space on CR.

**Results:**

Of the total 106 pneumothorax cases 80 patients had full lung expansion on CUS, the CD was located by CUS in 69 (86%), the CDI was 0.99 (0.88–1.06). 26 cases had a residual pneumothorax after drainage (24.5%), the CD was located by CUS in 31%, the CDI was 0.76 (0.6–0.93),*p* < 0.01. The risk ratio for a pneumothorax in a patient with not visible CD between the pleural layers on CUS and an associated low CDI on CR was 5.97, *p*˂0.0001. For the patients with a steep angle of inclination (> 50°) of the CD, the risk ratio for pneumothorax was not significant (*p* < 0.17). A continued air leak from the CD after drainage is related to the risk for a residual pneumothorax (RR 2.27, *p* = 0.003).

**Conclusion:**

Absence of a CD on CUS post drainage, low CDI on CR and continuous air leak significantly associate with residual occult pneumothorax which may evade diagnosis on an antero-posterior CR.

## Introduction

**Fig.1:**
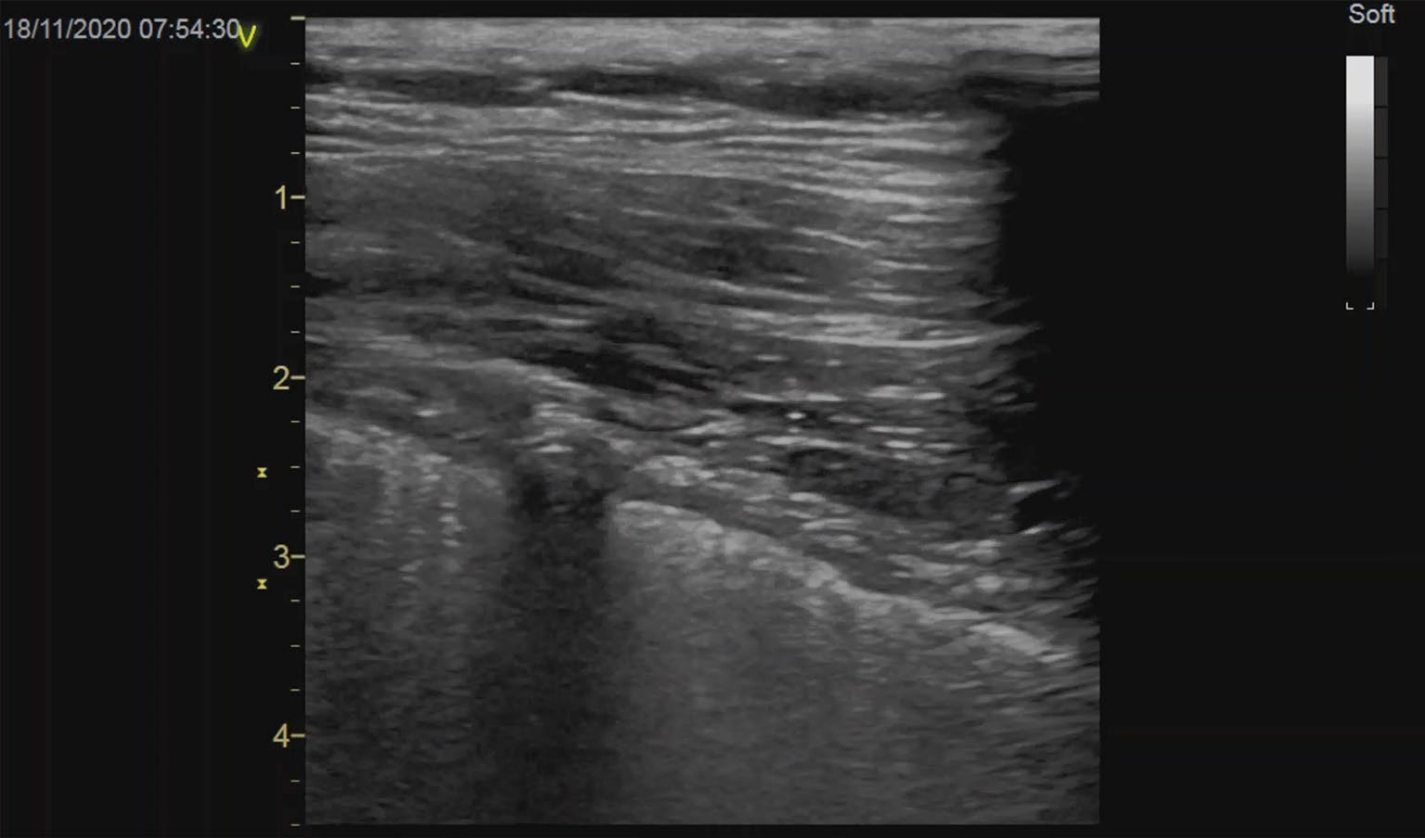
Chest ultrasound of the anterior chest wall in a Covid-19 ARDS patient after drainage of a pneumothorax due to barotrauma. The linear transducer depicts a chest drain in the transverse plane in between the enhancing visceral and parietal pleura

Chest ultrasound (CUS) has to certain degree replaced the chest X-ray (CR) and computer tomography (CT) scan in the critically ill with respiratory failure [1, 2]. A pneumothorax is a medical emergency especially in mechanically ventilated patients and in combination with Covid-19 ARDS, it is associated with poor outcomes [3]. Bedside CUS has become the “gold standard” for the early diagnosis of a pneumothorax [4]. CUS is also utilized to confirm full lung expansion after pleural drainage (Fig. 1). Full evacuation of a pneumothorax after chest drain placement may not be maintained due to increased occurrence of chest drain (CD) malposition [5]. This may potentially cause inadequate drainage, with ensuing ventral occult pneumothorax further limiting lung vital capacity in already severe lung disease and may also easily enlarge on an aggressive modality of intermittent positive pressure ventilation.

Serial CR imaging of patients with ARDS after CD insertion for pneumothorax remains a routine occurrence in most of the departments. However, the sensitivity of antero-posterior CR to detect pneumothorax is by 30% lower than the sensitivity of CUS [6, 7]. Hence, a CUS may be required to exclude a recurrent ventral pneumothorax that is occult on CR, or its recurrence on a previously fully expanded lung. During the COVID-19 outbreak, it is also feasible to minimize health care–patient interactions to only the essential procedures [8, 9]. Therefore, suspicion of a ventral pneumothorax on a CR can lead to further examination by CUS which may confirm or exclude the diagnosis.

**Fig.2:**
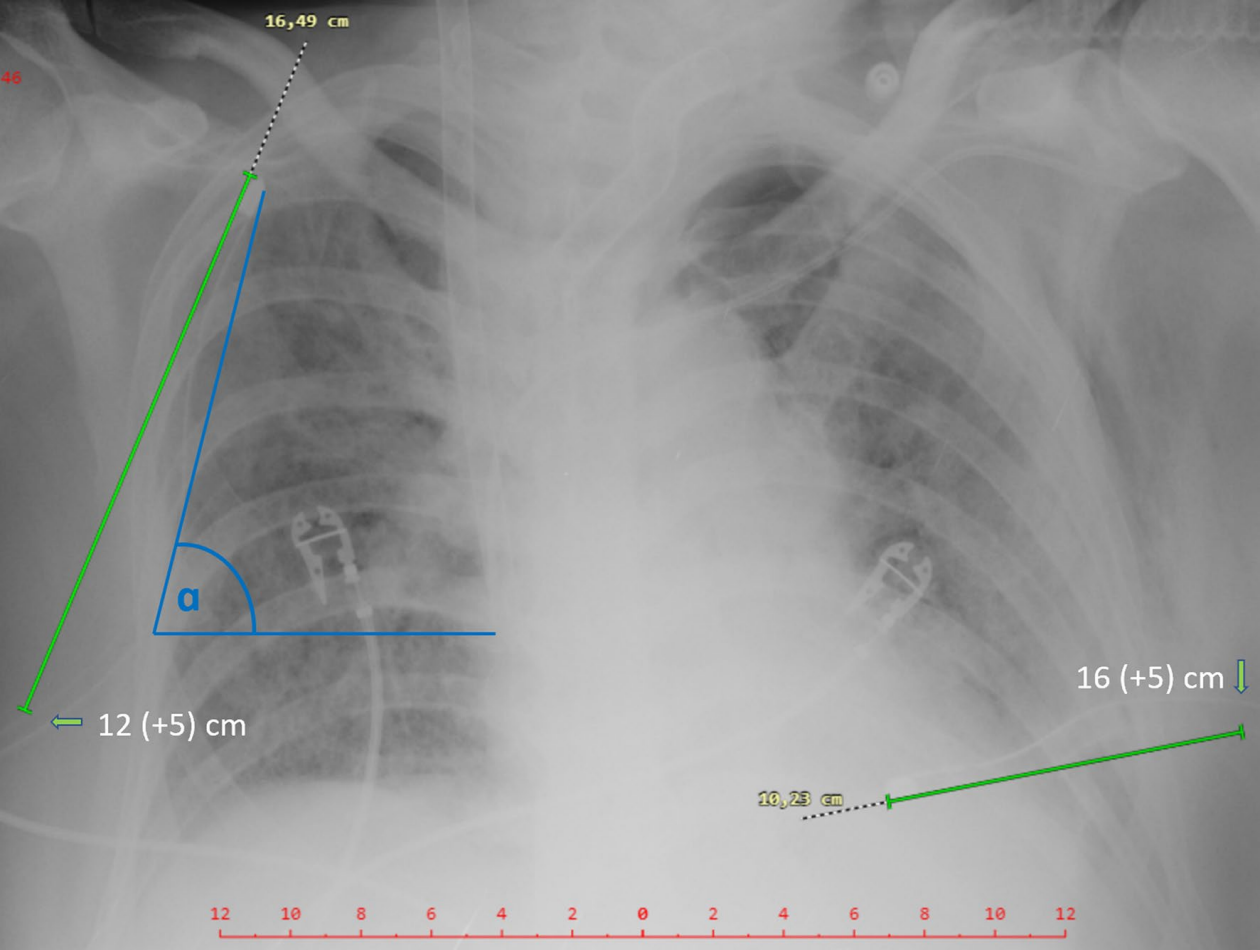
Patient on VV-ECMO with bilateral pneumothoraces has a steep ascending right CD (ɑ˃50°) with a CDI of 0.97 (16.49/12 + 5), and no signs of a pneumothorax on CUS of the right hemothorax. The left CD has a CDI of 0.49 (10.23/16 + 5 cm), foreshortening due to dorsal malposition and a left ventral pneumothorax on CUS (Fig. 5)

The position of a CD after drainage on available imaging may be used to detect adequate re-expansion of the lung. Our pilot study [10] utilizing chest CT showed that a greater foreshortening of the CD and a steep angle of inclination of the CD above the horizontal at chest entry taken from the CR (Fig. 2) should raise suspicion of CD migration from its optimal position under the anterior chest wall in a supine patient. The role of CD location by bedside imaging methods in the diagnosis of a pneumothorax has not yet been explored in a prospective study. Likewise, there is no standard protocol for using CUS to confirm the correct position of the CD.

The primary objective of our research was to evaluate how chest tube positioning, assessed via CUS and CR, may be associated with residual pneumothorax. CUS findings such as lung-point, absence of lung sliding, B-lines and the lung pulse were taken as a reference standard for the diagnosis of a pneumothorax. The hypothesis was that the absence of CD detection between the ventral pleural layers on the bedside CUS may be associated with signs of pneumothorax on CUS. These findings may be accompanied by the presence of a CD foreshortening and/or steep angle of inclination of the CD, which are parameters taken from CR. If confirmed, these new indicators alluding to CD malposition on CR following pneumothorax drainage may trigger further re-evaluation by CUS to confirm or exclude the diagnosis of an occult ventral pneumothorax.

## Materials and methods

**Fig.3:**
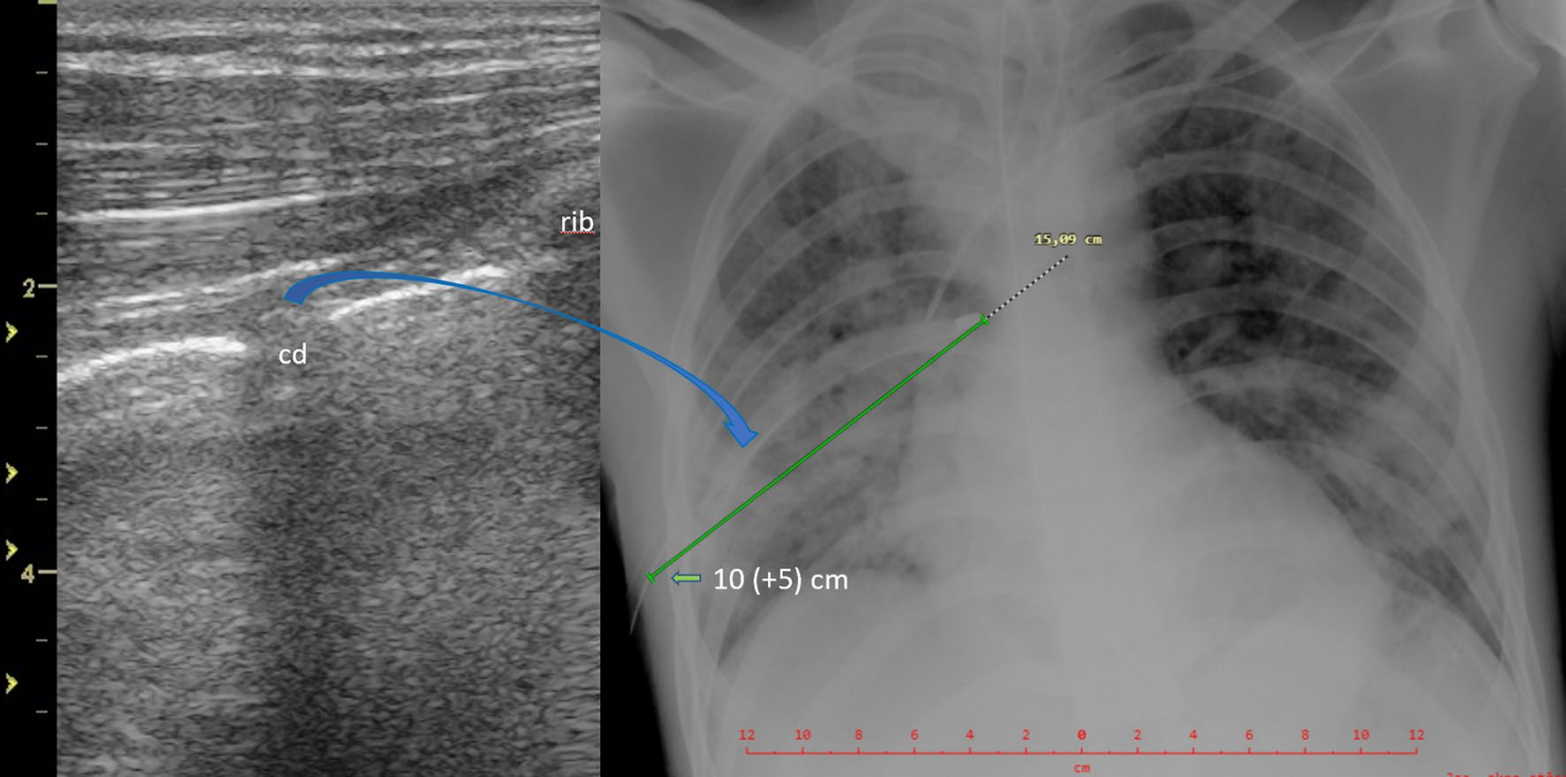
Patient with Covid-19 ARDS after CD insertion for a right ventral pneumothorax. The linear transducer shows the transverse plane of the CD between the enhancing pleural layers under the anterior chest wall next to the rib (left CUS, blue arrow towards the drain position on the right CR). In the same patient, the CDI (here 1.00) is equal to the length of the CD in the chest measured on CR (15.09 cm) divided by the depth of insertion of the CD read directly on a CD scale plus 5 cm (10 + 5 cm)

**Fig.4:**
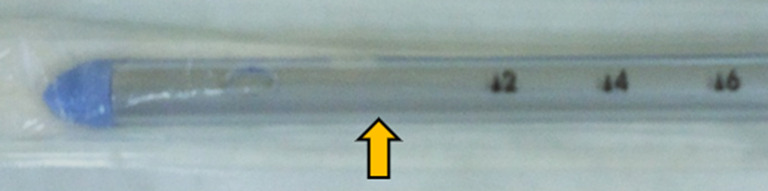
Tip of a 20F chest drain (Portex, UK). The distance between point zero of the scale (the first orifice) and the tip is 5 cm

We prospectively evaluated all patients with Covid-19 ARDS and a concomitant pneumothorax drained according to the standards from the safe triangle [11] for the presence of a residual pneumothorax on CUS, detection of a CD between the pleural layers on CUS, CD foreshortening and angle of CD inclination both taken from the bedside CR. All patients with large subcutaneous emphysema or anatomical drain malpositions were excluded. The systematic CUS examinations were performed in six regions on the right and left hemithorax [12]. The drainages were performed by intensivists using 16-20F CDs (Portex, UK) and utilizing the blunt forceps technique in the safe triangle [11]. The drains were pulled off the trocar under the anterior chest wall in the direction of the sternoclavicular joint and strictly without the trocar entering the pleural space [13, 14]. All drains were connected to a closed suction system with a negative pressure of −20 cmH_2_O. A pneumothorax was diagnosed on CUS according to the current standards [4] using the linear transducer (6–10 MHz, Vivid S6, VividS60 or Vivid I, General Electric) (Fig. 1). Foreshortening was estimated as a decrease in the chest drain index (CDI) which should ideally be close to 1 (Fig. 3). The CDI is equal to the length of the CD in the chest measured on an antero-posterior CR divided by the depth of insertion read directly on a CD scale plus 5 cm (Figs. 2, 3), which is the distance from the first drainage orifice to the tip of the CD (Fig. 4). The angle of inclination of the CD was measured as the angle between the horizontal line and the CD at pleural space entry on the CR (Fig. 2). The angle of inclination of the CD was judged to be higher or lower than 50° [10].

All analyses were performed using Statistica v.12 software. The normality of the data was tested using the Kolmogorov–Smirnov test and the statistical significance between the groups was tested using the Mann–Whitney U test for numerical variables and with the Chi-square test for categorical data. The numerical data are reported as medians and the interquartile ranges. The risk ratio for a pneumothorax on CUS was calculated in relation to the CR findings. A p-value below 0.05 was considered significant.

## Results

116 pneumothorax drainages (75 on the right, 41on the left) were performed and monitored in 88 patients (31 females, age 56.2 ± 19, APACHE II 22 ± 4, SOFA 9 ± 2.2) between March 2020–February 2022. 10 patients were excluded due to significant subcutaneous emphysema.

The etiologies of the pneumothorax were spontaneous on mechanical ventilation in 79 (74%), post-cannulation or due to thoracocentesis in 25 (24%) and after transbronchial biopsy in 2 (2%).

The results in groups with and without residual post-drainage pneumothorax are given in Table 1. Among the 80 cases with full lung expansion on CUS (no pneumothorax in the six zones of each hemithorax) the CD was located by CUS after drainage in 69 (86%). The median CDI was 0.99 (0.88–1.06), and the steep angle of inclination of the CD on CR (> 50°) was found in 10 patients (12.5%).

**Table1:** Comparison of the novel observed categorical parameters (CD location in %, its steep course in %, presence of an air leak in %) and continuous parameters (depth of CD insertion in cm, length of CD in chest in cm, CDI, all * medians and interquartile ranges) between groups with a full lung expansion on CUS (pneumothorax excluded in all lung fields) and group with a residual pneumothorax on CUS

Pneumothorax *n* = 106	Full lung expansion on post-drainage CUS (*n* = 80, 75%)	Residual pneumothorax on post-drainage CUS (*n* = 26, 25%)
Drain located on CUS	69 (86%)	8 (31%), (*p*˂0.0001)
Depth of drain insertion on CD scale (cm)*	12 (10–14)	12 (12–16), (n.s)
Length of CD in chest on antero-posterior CR (cm)*	16.1 (14.2–17.9)	13.3 (11.4–16.5), (n.s.)
CDI*	0.99 (0.88–1.06)	0.76 (0.6–0.93), (*p*˂0.01)
Steep ascending drain in chest on CR	10 (12.5%)	6 (23%), (n.s.)
Continued air leak from the drain	19/80 (24%)	14/26 (55%), (*p*˂0.003)

26 cases had a residual pneumothorax after drainage (24.5%), the CD was located by CUS in 8 of those (31%), the median CDI was 0.76 (0.6–0.93), *p* < 0.01, with the steep angle of inclination of the inserted CD on CR being observed in 6 patients (23%).

Of the 106 patients included, the CD was located in between the pleural layers in 77 patients, and 8 of those had a residual pneumothorax. In contrast, the CD was not located in 29 patients, of which 18 still had a post-drainage pneumothorax. The risk ratio for a pneumothorax in a patient with a CD that is not visible in the interpleural space on CUS (*n* = 29) and an associated low CDI on CR was 5.97, 95% CI [2.92–12.21], *p*˂0.0001, NNT 1.94.

For the 16 patients with a steep angle of inclination of the CD on CR of more than 50°, the risk ratio for a pneumothorax was not significant (RR 1.68, 95% CI [0.80–3.54], *p* < 0.17, NNT 6.55).

For the 33 patients with a continued air leak from the CD after drainage the risk of a residual pneumothorax is significant (RR 2.27, 95% CI [1.33–3.85], *p* = 0.003, NNT 3.32).

## Discussion

The observational study shows that a CD may be located on CUS under the anterior chest wall in 86% of patients after drainage and represents an important sign of successful pleural drainage with full lung expansion that has not been described so far (Fig. 1). However, failure to locate the CD carries a significant risk of a residual pneumothorax, which must be excluded on CUS [4]. The presence of a chest drain in between the pleural layers on CUS represents an additional important sign excluding a residual pneumothorax, particularly in the apical lung regions with limited lung sliding and lung pulse [15]. With its limitations given by interfering ribs the finding may help to exclude pneumothorax particularly in lung hyperinflation like COPD, bullous emphysema, post thoracic surgery and in patients with consolidated lungs on ECMO and a lung-protective mechanical ventilation.

**Fig.5:**
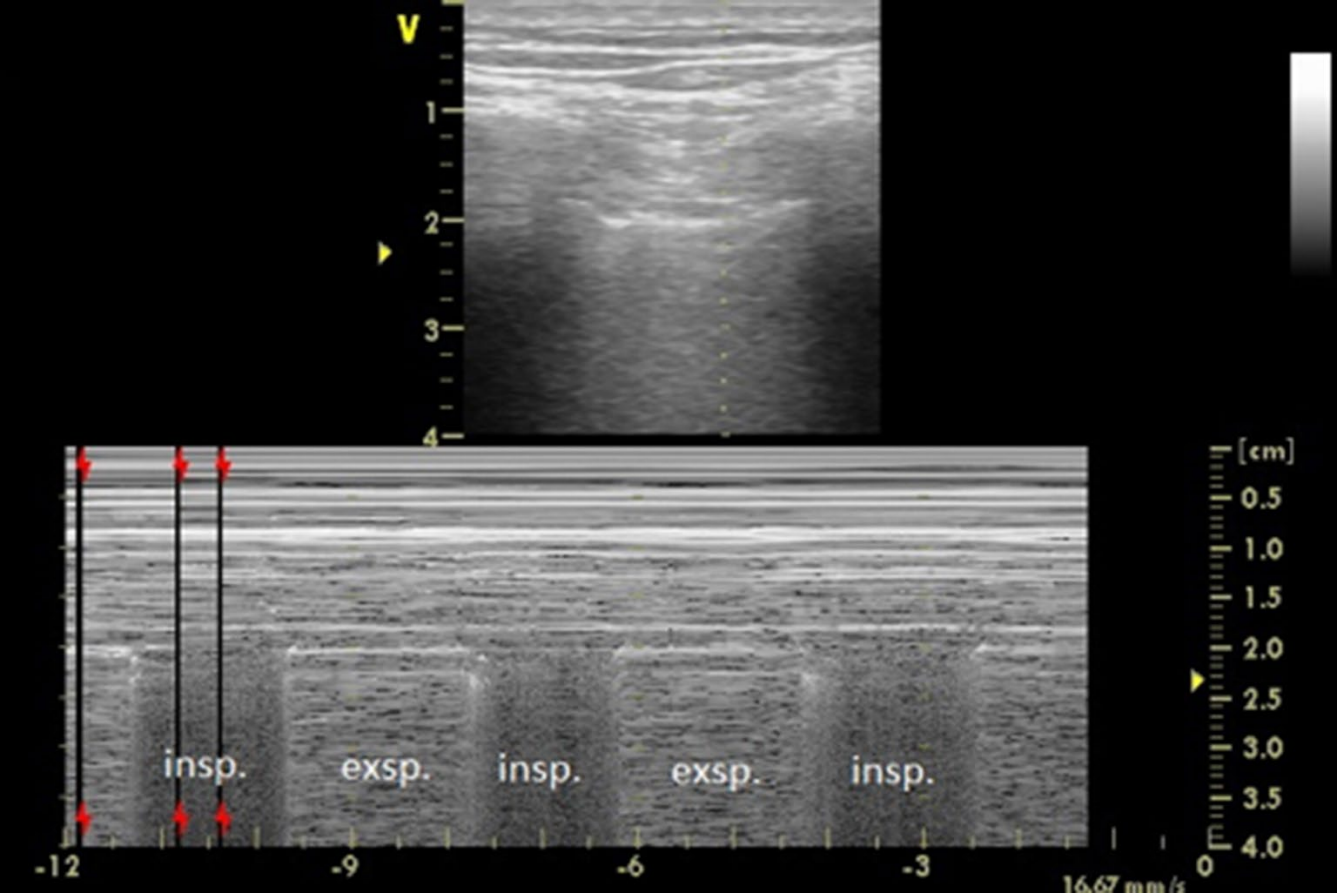
M-mode (linear transducer) during a respiratory cycle in anterior axillary line confirming ventral pneumothorax in a patient with low CDI. There is a bat sign of two ribs with the intercostal space between them. The seashore sign in inspirium changes with a pneumothorax (barcode sign) in exspirium (Fig. 4)

The degree of CD foreshortening on CR estimated with the help of the CDI implies a high risk of an occult ventral pneumothorax (Figs. 2, 5). Another clinical finding that warrants the exclusion of an occult pneumothorax is a continuous air leak from the inserted CD. In contrast to the conclusions of our retrospective study [10], the risk of a residual pneumothorax is likely not significant with a steep angle of inclination of the CD.

Limitations of the study include interobserver variability, which is less for pathologies of the pleural space as compared, for example, with CUS interrogation of the lung parenchyma [16]. A parallel course of the CD to a rib interfering with CUS was found in 10% of patients without any other CUS signs of a pneumothorax. Furthermore, the authors adhered to current recommendations [11] and excluded anterior CD insertions from the midclavicular access. Nonetheless, foreshortening of the CD and the CDI calculation may also apply to the ventral insertions of the chest drains. With high rates of CD migration after insertion, the authors see a practical application of locating a CD on CUS based on bedside CR parameters after pneumothorax drainage. However, a multivariate analysis combining these three significant findings (CD position on CUS, CDI, and air leak) could better assess their relative significance.

Estimation of CD foreshortening requires knowledge of the insertion spot and the depth of the CD in the chest. During radiology rounds, a low CDI on CR (Fig. 2) requires further investigation by CUS to rule out a residual pneumothorax occult on CR (Fig. 5). Co-operation between the person inserting the drain (intensivist) with the person interpreting the CR (radiologist or intensivist) may help to eliminate transport of a critically ill patient to the CT scan suite for a thoracic CT to exclude a residual pneumothorax, which is advantageous not only during pandemic of SARS-CoV-2.

## Conclusion

The presence of a CD on CUS post drainage rules out a presence of a pneumothorax and should be considered as an additional exclusion parameter. Its absence however, significantly associates with a residual pneumothorax post drainage which may evade diagnosis on an antero-posterior CR. A low CDI on CR and a continuous air leak from the drain should warrant a bedside CUS to exclude a recurrent occult ventral pneumothorax which may easily enlarge on intermittent positive pressure ventilation.

## Data Availability

The dataset analyzed during the study is available from the corresponding author upon request.

## Abbreviations

CUS: Chest ultrasound
CR: Chest X-ray
SARS-CoV-2: Severe acute respiratory syndrome-related coronavirus
ARDS: Adult respiratory distress syndrome
IPPV: Intermittent positive pressure ventilation
CD: Chest drain
CDI: Chest drain index
